# SG-APSIC1047: Patient instrument tracking system

**DOI:** 10.1017/ash.2023.107

**Published:** 2023-03-16

**Authors:** Nanthipha Sirijindadirat, Pattaraporn Noilek, Dujdarin Somboonsap

**Affiliations:** President, Central Sterilizing Services Association, Bankok, Thailand; Sirirajpiyamaharajkarun Hospital, Bagkok, Thailand; Sirirajpiyamaharajkarun Hospital, Bagkok, Thailand

## Abstract

**Background:** In the post–COVID-19 era, competency of healthcare workers is very important, and new technology is imperative. The central sterile supply department (CSSD) staff must also improve their response when a patient infection is reported. The sterilization process is more important than ever. **Objectives:** We sought to simplify surveillance to act faster, to reduce the time to obtain patient data, and to eliminate nonvalue stream mapping in the workflow process in order to prevent patient harm and strengthen our infection prevention and control efforts. **Methods:** The CSSD staff met with an IT developer to determine requirements for an electronic surveillance program. Before this intervention, we scanned hard copies of patient records and stored them in a folder on a computer on a daily basis. These data were difficult to search, monitor, and display, and this method wasted time in locating patient data. The IT developer designed a program to track patients and instruments. The program collected data regarding the patient’s surgery, instruments used, and monitoring information. With the help of the IT developer, the CSSD staff tested and tweaked the new platform until accuracy and usability were achieved. Staff were trained on the use of the new system before it was implemented. **Results:** This project yielded simplified surveillance that improved the infection prevention process, reduced potential patient harm, and strengthened the ability of the IPC team to analyze and act on data. **Conclusions:** A simple surveillance system for tracking patients and instruments used assists both CSSD and IPC teams. This system assures the performance of sterilization procedures. When adverse events occur, patients who used these devices are tracked, and an analysis is performed to identify and implement improvements.

